# Reasons and Barriers for Using a Patient Portal: Survey Among Patients With Diabetes Mellitus

**DOI:** 10.2196/jmir.3457

**Published:** 2014-11-25

**Authors:** Maaike CM Ronda, Lioe-Ting Dijkhorst-Oei, Guy EHM Rutten

**Affiliations:** ^1^Julius CenterJulius Center for Health Sciences and Primary CareUniversity Medical Center UtrechtUtrechtNetherlands; ^2^Internal MedicineDepartment of Internal MedicineMeander Medical CenterAmersfoortNetherlands

**Keywords:** diabetes mellitus, telemedicine, Internet, electronic health record, cross-sectional studies, patient preference, patient access to records

## Abstract

**Background:**

The use of a Web portal for patients with diabetes mellitus to access their own personal health record may result in improved diabetes outcomes. However, the adoption by patients is slow. This may be caused by patient characteristics, but also by the content, layout, and promotion of the portal. Detailed knowledge about this could help increase patients’ participation in Web portals.

**Objective:**

The aim was to study the opinions of patients with diabetes and identify perceived barriers to using a Web portal to optimize its use.

**Methods:**

We conducted a survey among patients with type 1 and type 2 diabetes mellitus from 62 primary care practices and 1 outpatient hospital clinic in the central area of the Netherlands who all used the same electronic health record with a Web portal. Questionnaires about patient characteristics, opinions about reasons for use or nonuse, and about portal content were sent to 1500 patients with a login and 3000 patients without a login to the Web portal. Patient groups were stratified according to login frequency. Demographic and diabetes-related variables were analyzed with multivariable regression analysis.

**Results:**

The total response rate was 66.63% (2391/4399); 1390 of 4399 patients (31.60%) were eligible for analysis. There were 413 regular users (login frequency more than once) and 758 nonusers (no login). Most nonusers (72.4%) stated that the main reason for not requesting a login was that they were unaware of the existence of the portal. Other barriers reported by patients were disinterest in managing their own disease (28.5%, 216/758) and feelings of inadequacy with the use of computers and Internet (11.6%, 88/758). Patients treated by a general practitioner were more frequently nonusers compared to patients treated by an internist (78.8%, 666/846 vs 28.3%, 92/325; *P*<.001) and more users than nonusers became aware of the Web portal through their physician (94.9%, 392/413 vs 48.8%, 102/209; *P*<.001). Nonusers perceived specific portal content as not as useful as regular users did, especially access to laboratory values (71.7%, 383/534 vs 92.3%, 372/403), rereading clinic visits (61.3%, 320/522 vs 89.6%, 360/402), e-messaging (52.0%, 262/504 vs 74.6%, 299/401), and uploading results to the glucose diary (45.3%, 229/506 vs 74.0%, 288/400; all *P*<.001).

**Conclusions:**

Our study shows that unawareness of the patient portal is the main barrier of enrollment. Users and nonusers perceive the usefulness of the portal differently and do not have the same recommendations for additional functionalities. To increase patients’ participation in a Web portal, the unawareness of its existence and its possibilities need to be addressed by their health care professionals.

## Introduction

The use of eHealth in disease management has been studied, especially in chronic diseases such as diabetes mellitus. In these studies, the focus was on patient Web portals where patients have access to their medical health record and can use the Web portal for communication with their health care provider. The use of a Web portal has several benefits. It can enhance communication between patient and health care professionals [1], allow patients to play a more active role in their own treatment and self-management [2], increase self-efficacy [3], and patients can feel that other nonacute concerns are valued because of an email function [4]. The use of Web portals shows promising results in diabetes outcomes, such as improved HbA_1c_, blood pressure, weight, and cholesterol levels [5-9]. With the growing number of people with diabetes mellitus worldwide [10], the use of patient portals for diabetes management becomes more important to cope with the burden on health care.

However, the adoption of Web portals is slow by both patients [11] and health care professionals [12]. We previously showed that patient characteristics play an important role in nonadoption [13]. Simply promoting eHealth is ineffective without addressing the differences in patient characteristics.

In the Netherlands, 96% of all inhabitants have access to Internet. Men and women have equal access and more than 95% of people up to age 65 years have access; the access rate is lower (81%) for people older than that age. Access ranges from 90% in lower education groups to 99% in the groups with the highest education. Of the people with Internet access, 87% use it daily [14]. Therefore, Internet access itself should not be a barrier for use of patient portals by most patients with diabetes mellitus.

For both patients and providers, there are several barriers in the adoption of a Web portal. Health information privacy and security are major concerns [15]. In addition, the use of medical terms and abbreviations [15,16] and problems arising due to the design [11], such as navigational problems and unmet expectations about functionality, may also play a role. There is a difference in the potential and actual usefulness of certain features of a Web portal [17]. Before using a Web portal, patients have certain expectations about how the portal may help them with their disease management and which features may be useful for them. These opinions may change when patients actively use the portal.

However, it remains unclear what reasons patients with diabetes have for using a Web portal or not. Previous research has not fully considered the steps that need to be taken before patients decide whether a patient portal can be of personal use. If we want to increase the involvement of patients in their own treatment, the barriers for using a Web portal must be addressed. More information is needed about the opinions that patients have when deciding to login for a Web portal or not and about their first experiences with its use. With this knowledge, the initial barriers of using a Web portal could be reduced. This study aims to study the opinions and barriers of patients with diabetes to request a login and to use a patient Web portal. The following research questions were addressed:

1. In what respect do regular users and nonusers of the portal differ?
2. What are the reasons for (or not) requesting a login?
3. How did patients become aware of the portal?
4. Are there any differences in perceived usefulness of the portal between users and nonusers?
5. Are there recommendations for new functionalities?

## Methods

### Design

We conducted a survey among a sample of 12,793 patients with diabetes by randomly selecting patients aged 18 to 85 years and asking 1500 patients with a login to the Web portal and 3000 patients without a login to participate. Patients were sent a set of questionnaires and a reminder letter twice if necessary. Patients who did not want to participate were asked to state the reason. The survey was approved by the Medical Research Ethics Committee of the University Medical Center Utrecht (protocol number 11-296/C).

### Setting

**Figure 1:**
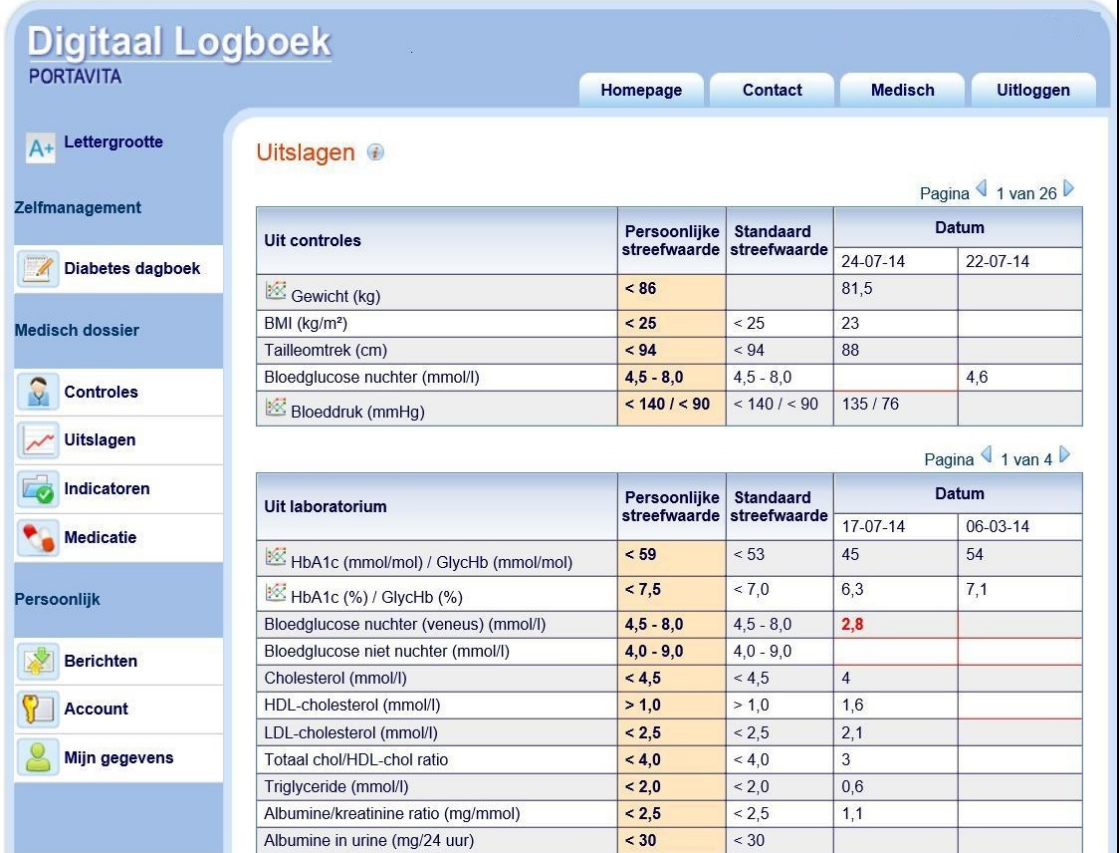
Screenshot of the laboratory results and treatment goals of the patient Web portal.

Primary care practices and the regional hospital joined together in an organization called “Diamuraal” to coordinate the care of patients with diabetes in a defined geographical area in the center of the Netherlands. Currently, Diamuraal comprises 62 independent primary care practices and 1 hospital outpatient clinic. All physicians and nurses who participate in the care of patients with type 1 and type 2 diabetes in Diamuraal use the same electronic health record and patients can request a login to access their personal electronic health records. When a patient wants access to the Web portal, he or she needs to sign a registration form which the physician has to cosign. The portal is called “Digitaal Logboek” and was developed by Diamuraal and a private company (Portavita). Patients have access to their diabetes-specific medical records, including information provided by their physician during medical consultation, such as physical examination, laboratory results, problem lists, and treatment goals (Figure 1). Laboratory results are accessible as soon as the laboratories report them in advance of a medical consultation. The Web portal also provides access to general diabetes information and to an overview of all personal diabetes-related examinations and consultations that are needed and/or scheduled. Patients can import and upload the glucose levels measured at home and contact their physician or diabetes nurse through secured electronic messaging. The portal is supplementary; patients who do not request access still receive diabetes care according to the Dutch guidelines. At the start of our survey, 12,793 patients with diabetes were treated in Diamuraal, of whom 9791 (76.53%) never requested a login.

### Measures

We collected information through a set of questionnaires and by extracting data from the electronic health records. Patient characteristics were obtained from the patients’ electronic health records (age, gender, type of diabetes, duration of diabetes, setting of diabetes treatment, HbA_1c_, and total cholesterol) and from the questionnaires (educational level, ethnicity, living status, employment, medication, smoking, drinking, fluency in the Dutch language, and access to computer and Internet). We did not take blood pressure into account because it was not a determinant for portal use [13].

### Questionnaires

We designed 2 separate questionnaires: 1 for patients with a login and 1 for patients without a login. They were based on characteristics found previously on the use of eHealth in literature [18,19].

The questionnaire for patients with a login contained multiple choice questions about (1) reasons for requesting a login (influence in disease and management of disease, to reread information at home, others thought it would be useful, discontent with current care, other); (2) the way people were informed about the portal’s existence (by a health care provider, a poster, an information pamphlet, through friends or relatives, other); (3) the frequency of portal use (from daily to monthly); (4) the duration of portal use (from less than 15 minutes to more than 1 hour); and (5) the person who added the information to the portal (the user/self, family, friends, or others).

The questionnaire for patients without a login contained questions about their awareness of the patient Web portal and, if applicable, how people were informed about its existence (see above) as well as the reasons for nonuse (all yes/no questions). There was room for free text as well.

Both questionnaires contained questions about the use of the Internet for other purposes than the Web portal, with regard to frequency and duration and the use of the Internet for searching information about diabetes (from never to monthly). The 9 questions about the perceived usefulness of specific portal components were answered on a 5-point Likert scale ranging from very important to unimportant.

The final question regarded possible improvements to the portal. All recommendations were scored on a 5-point Likert scale ranging from very important to unimportant. There was room for free text.

Questions about specific portal components and the question about recommendations regarding possible improvements were, in case of nonusers, addressed as how nonusers expected the usefulness of that particular component to be.

In addition to these specifically designed questionnaires, the set of questionnaires contained additional validated questionnaires, including the Problem Areas in Diabetes (PAID) questionnaire to measure diabetes-specific distress [20,21], the Diabetes Treatment Satisfaction Questionnaire (DTSQ) to measure satisfaction with diabetes treatment [22], the Diabetes Management Self-Efficacy Scale (DMSES) to measure self-efficacy [23], and the Brief Diabetes Knowledge Test (BDKT) to measure diabetes knowledge [24,25].

### Statistical Analysis

Reason for regular use or nonuse, the answers about content and usefulness of the portal, and about the recommendations were expressed as percentages. The answer categories useful and very useful were combined. The question about reason for nonuse was misread by some patients. We asked for the main reason (1 reason) why a patient did not request a login and provided multiple answers. A total of 59 patients gave more than 1 reason. We used all these answers in the analysis.

We compared patients who requested a login and used it 2 or more times (regular users) and patients who did not request a login at all (nonusers). We decided to perform the analysis only on the regular users instead of all patients with a login because we wanted to compare the patients without a login to a group of patients with actual experience with the patient portal. Based on previous research, we considered the group of nonusers too different from patients who had requested a login but never logged in or logged in only once, the so-called “early quitters.” Indeed, early quitters differed from nonusers: they were younger (mean 61.9, SD 12.7 years vs mean 64.7, SD 10.0 years; *P*=.001), more often male (63.9%, 140/219 vs 56.5%, 428/758; *P*=.049), and had a higher educational level (39.4%, 84/213 vs 28.2%, 519/723; *P*=.002).

Age and gender of nonparticipants (patients who responded but declared that they did not want to participate) and nonresponders (patients who did not respond to the invitation to fill out the questionnaire) of the study are described elsewhere [13].

We used chi-square tests for all categorical variables and unpaired *t* tests for all normally distributed continuous variables and Mann-Whitney tests for nonnormally distributed continuous variables. Categorical variables were expressed as numbers with percentages and continuous variables as means with standard deviation (SD) or with median and interquartile range (IQR) when not normally distributed. Multivariable logistic regression analysis, using the enter method, was used to determine the adjusted association between patient characteristics and not requesting a login. We used a *P* value of <0.2 in the univariable analysis to select variables for further multivariable analysis. These determinants were expressed as odds ratios (ORs) with corresponding 95% confidence intervals (95% CI). Data was analyzed using SPSS for Windows version 20 (SPSS Inc, Chicago, IL, USA).

## Results

### Overview

From the 4500 questionnaires, 101 were not answered because 33 patients died and 68 had incorrect contact information. From the remaining patients, 2931 (66.63%) responded; 1541 of these 2931 patients (52.59%) declared that they did not wish to participate. In total, 1390 (31.60%) patients were eligible for analysis (“participants”) because they returned a completed questionnaire and signed a consent form. Their mean age was 63.9 (SD 12.2) years (nonparticipants: mean 64.5, SD 13.8 years; *P*=.11) and 826 of 1390 (59.42%) were male (nonparticipant group: 1539/3009, 51.15% male patients; *P*<.001). Of the 1390 participants, 632 (45.47%) had a login and 758 (54.53%) did not (“nonusers”).

### Differences Between Nonusers and Regular Users

The login frequency of the patients with a login was a mean 10.4 (SD 23.0) times and 413 of 632 (65.3%) patients accessed the patient Web portal 2 or more times (“regular users”). The latter category differed in many characteristics from nonusers (Table 1). Of the 94 patients with type 1 diabetes, only 13 (14%) were nonusers, whereas 745 of 1077 (69.17%) patients with type 2 diabetes were nonusers (*P*<.001). There was also a difference in treatment setting: 666 of 846 (78.8%) patients treated by a general practitioner were nonusers, whereas only 92 of 325 (28.3%) patients treated by an internist were nonusers (*P*<.001).

The use of the Internet differed between both groups: 321 (77.9%) of the 413 regular users used the Internet daily versus 346 (67.6%) of the 512 nonusers with Internet access (*P*<.001). When using the Internet, 184 (44.6%) of the 413 regular Web portal users were online for more than an hour per day compared with only 140 (27.3%) of the 512 nonusers (*P*<.001). Furthermore, 206 (51.1%) of the regular users declared that they used the Internet for searching for information about their disease compared with only 126 (25.4%) of the nonusers (*P*<.001).

Of the 413 regular users, 328 (79.4%) patients declared that they were the main user of the Web portal themselves and 79 (19.1%) declared that someone else had access to the Web portal and usually accessed the portal. Of the 758 nonusers, 162 (21.4%) patients stated that they would consider using the Web portal if someone would help them, 262 (34.6%) did not know if they would use the portal if someone would help, and 293 (38.7%) would not consider using the portal even if someone would help.

Multivariable analysis showed that increasing age and smoking were associated with not using the Web portal. On the contrary, a higher educational level, treatment by an internist, using insulin, polypharmacy, better diabetes knowledge, and more hyperglycemic episodes were less likely to be associated with not using the Web portal (Table 2).

**Table 1:** Characteristics of the study participants (N=1171).

Patient characteristics		Regular users (n=413)	Nonusers (n=758)	*P*
Age (years), median (IQR)		60.2 (51.3-67.5)	68.1 (60.7-75.3)	<.001
Gender (male), n (%)		259 (62.7)	428 (56.5)	.04
Caucasian (yes), n (%)		383 (93.6)	652 (89.3)	.02
Educational level (high), n (%)		188 (46.2)	204 (28.2)	<.001
**Work status, n (%)**				<.001
	Paid job	193 (47.1)	157 (21.1)	
	Retired	153 (37.3)	479 (64.5)	
	Other	64 (15.6)	107 (14.4)	
Living arrangement (alone), n (%)		65 (15.9)	193 (25.9)	<.001
Fluency in speaking Dutch (yes), n (%)		407 (99.3)	695 (93.0)	<.001
Access to computer (yes), n (%)		413 (100)	525 (70.5)	<.001
Access to Internet (yes), n (%)		413 (100)	516 (84.7)	<.001
**Treatment setting, n (%)**				<.001
	General practitioner	180 (43.6)	666 (87.9)	
	Internist	233 (56.4)	92 (12.1)	
**Type of diabetes, n (%)**				<.001
	Type 1	81 (19.6)	13 (1.7)	
	Type 2	332 (80.4)	745 (98.3)	
Duration of diabetes (years), median (IQR)		11.3 (5.5-17.4)	7.4 (3.7-11.4)	<.001
**Blood glucose lowering medication, n (%)**				<.001
	None	21 (5.1)	91 (12.1)	
	Oral	131 (31.7)	507 (67.2)	
	Oral and insulin	126 (30.5)	93 (12.3)	
	Insulin	135 (32.7)	64 (8.5)	
Polypharmacy (yes)		204 (52.7)	277 (43.6)	.02
HbA_1c_ (mmol/mol), median (IQR)		54.0 (48.0-62.0)	49.0 (44.0-56.0)	<.001
Total cholesterol (mmol/L), mean (SD)		4.4 (1.0)	4.5 (1.0)	.35
Smoking (yes), n (%)		47 (11.5)	116 (16.4)	.03
Drinking alcohol (yes), n (%)		208 (52.8)	294 (42.7)	.004
**Validated questionnaires,** ^a^ **mean (SD)**				
	PAID	31.0 (11.8)	27.2 (11.2)	<.001
	DMSES	80.7 (15.5)	72.9 (18.0)	<.001
	BDKT standard	78.7 (14.7)	62.4 (20.0)	<.001
	BDKT insulin	61.4 (20.6)	42.2 (21.5)	<.001
	DTSQ status	30.2 (5.0)	30.8 (5.5)	.10
	DTSQ hyperglycemic episodes	2.7 (1.9)	1.6 (1.7)	<.001
	DTSQ hypoglycemic episodes	2.0 (1.7)	1.1 (1.5)	<.001

^a^ PAID: Problem Areas in Diabetes Questionnaire; DTSQ: Diabetes Treatment Satisfaction Questionnaire (with treatment satisfaction status, perceived hypoglycemic and hyperglycemic episodes) ; DMSES: Diabetes Management Self-Efficacy Scale; BDKT: Brief Diabetes Knowledge Test (one with standard items and one with only insulin-related questions).

**Table 2:** Independent determinants of nonusers compared to users.

Independent determinant		OR (95% CI)	*P* value
Age		1.04 (1.00-1.08)	.03
Educational level (high)		0.59 (0.36-0.95)	.03
Treatment setting (internist)		0.27 (0.14-0.54)	<.001
**Blood glucose lowering drugs**			
	None	0.59 (0.21-1.63)	.31
	Oral	Reference	
	Oral and insulin	0.33 (0.15-0.70)	.004
	Insulin	0.31 (0.12-0.78)	.01
Polypharmacy (yes)		0.58 (0.36-0.95)	.03
Smoking (yes)		2.53 (1.30-4.91)	.006
Diabetes knowledge (standard)		0.98 (0.96-0.99)	.008
DTSQ (hyper)		0.79 (0.68-0.92)	.002

### Reasons for Requesting or Not Requesting a Login

The main reason for not requesting a login was that 549 of 758 (72.4%) patients were not aware of the portal’s existence. Another 216 of 758 (28.5%) stated that the main reason for not requesting a login was that they preferred to leave the disease management to the physician (Table 3).

The reasons for requesting a login among the regular users were to reread information of the consultation at home (312/413, 75.5%), the feeling that the portal use would give them influence on their disease and treatment (132/413, 32.0%), the fact that the physician or someone else thought the Web portal could be useful for them (74/413, 17.9%), dissatisfaction with the current care (2/413, 0.5%), and other reasons (27/413, 6.5%).

**Table 3:** Reasons for not requesting a login to the patient Web portal.

Reasons for not requesting a login	Nonusers, n (%) (n=758)
Was not aware that the portal existed	549 (72.4)
Prefers to leave disease management to physician	216 (28.5)
Feels inadequate with computer or Internet	88 (11.6)
No access to computer or Internet	62 (8.2)
Web portal is difficult to use	58 (7.7)
Privacy reasons	46 (6.1)
Concern for less personal attention by physician	48 (6.3)
Physician/other advised against portal use	20 (2.6)
Language barriers	18 (2.4)

### How Patients Became Aware of the Web Portal

Of the 209 patients without a login who stated they were aware of the existence of the portal, 102 (48.8%) knew about the portal because their health care provider told them. In comparison, 392 (94.9%) of the 413 regular users were informed about the portal by their health care provider (*P*<.001). Other sources of information about using the Web portal were posters in the clinic waiting area (nonusers: 10/209, 4.8%; regular users: 7/413, 0.7%; *P*<.001), a pamphlet (nonusers: 4/209, 1.9%; regular users: 15/413, 3.6%; *P*=.24), friends or relatives who used the portal themselves (nonusers: 20/209, 9.6%; regular users: 4/413, 1.0%; *P*<.001), and other reasons (nonusers: 29/209, 13.9%; users: 9/413, 2.2%; *P*<.001).

### Perceived Usefulness

**Figure 2:**
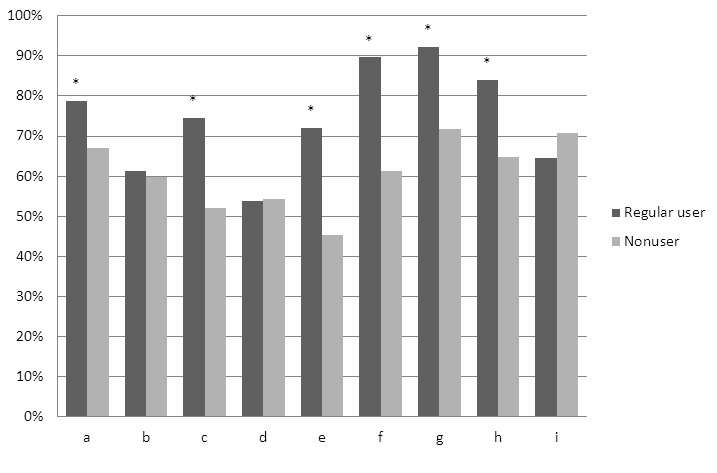
Differences between regular users (n=413) and nonusers (n=758) regarding the perceived usefulness (very useful or useful) of the content items of the patient Web portal. (a) overview of upcoming consultations, (b) summary of all health care physicians involved in treatment, (c) e-messaging, (d) general information about diabetes, (e) using the portal to upload the glucose levels measured at home, (f) rereading medical record after consultation, (g) access to laboratory values and treatment goals, (h) a summary of all consultations (history and future), (i) overview of medication. * P<.001.

Regular users perceived the usefulness of specific portal content in a different way compared to nonusers (Figure 2). Users perceived access to the laboratory values with treatment targets, the possibility of rereading clinic consultations, and having a summary of all controls as the most useful features of the portal. We asked the nonusers if they could speculate on the possible usefulness of portal features for their own disease management. They suggested a summary of upcoming consultations and a summary of their medication to be the most useful parts of a Web portal.

### Recommendations About Functionalities Added to the Web Portal

**Figure 3:**
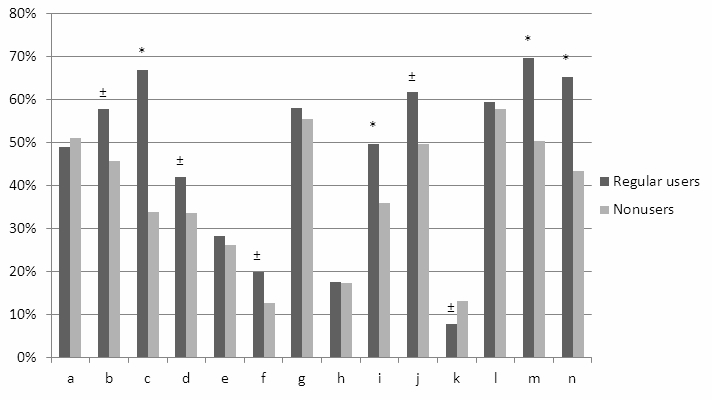
Differences between regular users (n=413) and nonusers (n=758) regarding usefulness of functionalities (very useful or useful) that could be added to the Web portal. (a) Automatic signal to physician when uploading glucose diary, (b) automatic upload from glucose meter to portal, (c) adding insulin units to glucose diary, (d) links to websites with information about diabetes, (e) links to websites with lifestyle interventions, (f) portal on mobile device, (g) request for medication refills, (h) forum functionality, (i) printing functionality, (j) news sites about diabetes, (k) information in different languages, (l) information about medication and side effects, (m) reminder function about upcoming consultation, (n) using the portal for scheduling a consultation with physician. * P<.001; ± P<.05.

Regular users and nonusers appreciated additional functionalities that could improve the Web portal differentially (Figure 3). Regular users wanted to be able to add their injected insulin units to the glucose diary and to use the portal for supportive care, such as scheduling an appointment and receiving reminders about upcoming consultations. Nonusers felt that a diabetes Web portal could benefit mostly from more information about medication and side effects and they wanted to use the portal for medication refills. Overall, regular users scored more possible features as useful or very useful than nonusers did except for information provided in different languages.

## Discussion

The main reason for patients with diabetes not requesting a login for a patient Web portal was that they were not even aware of its existence. This was previously found in a smaller group (3 of 13 respondents) of patients with diabetes mellitus type 2 [26]. Earlier studies have provided information on difficulties in usability [11,15,16] and reasons for not using the portal after receiving a login [27], but not on reasons for use or nonuse before requesting a login. In our study, it seems obvious that many health care providers, especially in the primary care setting, often did not communicate the possibility of using the shared electronic health record with their patients clearly enough. We can only speculate about the reasons. In the Netherlands, more than 99% of the primary care physicians and nurses work with an electronic medical system. However, it might be that they have not included a communication protocol about the Web portal for their patients with diabetes; they may not want to share data in a Web portal; they may have assumptions about capabilities, skills, and wishes of their patients that do not enhance the Web portal’s promotion [28]; or that they may not be satisfied with the Web portal itself [29]. Whatever the reasons, before trying to get a Web portal used by a substantial number of patients with diabetes, such a Web portal should be discussed in detail about requirements with all diabetes care providers. The same held true in telemonitoring of patients with heart failure; without transparent and predefined criteria of user requirements, health professionals expectations did not meet actual experiences, leading to disappointment [30]. Another possibility for the patients’ ignorance of the Web portal is that health care providers did communicate about the portal with their patients, but the latter did not recollect the physician telling them about it, perhaps because they did not understand the topic.

In a previous study, one of the main obstacles of enrollment in a general Web portal was that a quarter of the patients did not remember discussing the portal with their providers [31]. In that study, even despite remembering a discussion about the portal, another 63% of patients did not attempt to enroll mainly due to lack of motivation and negative attitudes toward the patient portal [31]. In the 6 years Diamuraal has been in use, 76.5% of the patients who could request a login never did. This percentage is more or less similar with other patient portals. In the literature, the actual percentage of users is approximately 32% to 37% for patients with chronic diseases such as diabetes [31,32]. In a general population, there is even less inclination to activate an account [11]. Better strategies have to be found to inform patients about a Web portal, how to request a login, and what benefits a patient portal may offer. One study in the general population found a threefold increase in Web portal enrollment with the use of aggressive marketing strategies, defined as using more than 5 different means of recruitment, including posters in the waiting area and onsite enrollment with a computer kiosk [33], illustrating the importance of the health care provider.

Some patients who did not request a login did so because they preferred to leave the disease management to the health care provider. On the other hand, regular users wanted to reread the information given by the diabetes care provider at home and they felt the portal gave them influence in the management of the disease. This illustrates the difference in opinions about who should be responsible for the management of the disease. In a previous study, we found that only 62% of patients with diabetes agreed to take full responsibility for their disease [34]. Therefore, we cannot expect that all patients will use and benefit from a diabetes patient Web portal.

Fear for privacy and security of the Web portal did not seem to pose a large barrier in our study in contrast to previous studies [35]. After patients have received access to their health care record, worries about the security may drop, for example, from 47% before to 4% after login when patients were reassured about the use of passwords and unique login numbers [15]. Health technology developers have to warrant the patient’s privacy without making the login process a barrier on itself and diabetes care providers should address the fears by informing patients about security measures.

Nonusers were older, had lower education [26,36], and had less diabetes knowledge. Diabetes care providers need to pay extra attention to this group of patients to help them becoming familiar with a different approach to diabetes care. At least one-fifth of the nonusers stated that they would use the portal if someone else could help them and another third of participants would at least consider using it. Many regular users stated that someone else used the portal as well to read the information provided by the physician. This access to the Web portal by family members has been shown to be effective and desirable in cardiac surgery [37] and in pediatric patient portals [27]. For all patients, the joint use of the Web portal by the patients themselves and a family member or friend should be discussed.

Patients can encounter difficulties in navigating through a portal to find the information they seek and have problems with interpretation of data [16,29,38]. This study does not provide any information in this respect because we sent questionnaires to nonusers who never logged in to the portal and could not comment on its attractiveness and ergonomics. However, if we want more patients using a portal, this is a concern that needs to be addressed. We are currently studying the influence of design and ease of use of the portal on persistent use or early discontinuation.

Not only actual barriers can prevent patients from requesting a login; nonusers perceived the usefulness of a patient Web portal differently compared to users. Although more users found the features that helped them with their disease control (very) useful, such as laboratory results and treatment goals, fewer nonusers scored those features as useful. Before using a Web portal, patients have certain expectations about which features are useful for them with regard to disease management and these expectations and opinions may change after actively using it [17]. The results of our study are another illustration of the fact that we need to inform patients better about what a patient portal can mean for them. To interpret our results correctly, we should keep in mind that we analyzed patients who had logged in 2 or more times. These regular users have other demographics than patients who cease to use the portal in an early stage and are not comparable to regular users or nonusers. Although there are differences between patients with type 1 and type 2 diabetes in requesting a login to the Web portal [13], we did not distinguish between type 1 and type 2 diabetes in the present study.

To our knowledge, this is the first study about the barriers of the use of a Web portal for patients with diabetes, before and during its use. The Web portal is used by patients with both type 1 and type 2 diabetes in primary care and also in secondary care. The Web portal under study has been used for 6 years, which underpins the relevance of the patients’ opinions. Furthermore, we studied a large group of nonusers of a patient Web portal for diabetes mellitus, not previously done in the literature.

However, there are some limitations. The first is due to the design of the questionnaire. Several patients gave multiple reasons for not requesting a login instead of 1 main reason, whereas other patients only gave 1 answer as per the instructions of the questionnaire. We are aware that patients can have multiple reasons for not requesting a login, but because most participants were careful in following instructions, they did not mention other reasons even if there were any. This means that our results are likely to be an underrepresentation of reasons for not requesting a login.

Secondly, there was a response rate of 66%; 31.6% of all people who were sent a questionnaire were eligible for analysis. This is comparable with other studies in this area [39,40]. Our participants did not differ in age from nonparticipants, but they were more frequently male. Gender was not a determinant for being a nonuser; therefore, the selective participation will not have influenced our outcomes.

Our study showed that unawareness of the patient portal is the main barrier of enrollment. All patients who were aware of the existence of the Web portal were made aware by their health care provider. Users and nonusers perceive the usefulness of the portal differently and do not have the same recommendations for additional functionalities. Currently, the Web portal is not communicated at all or not communicated clearly enough by health care providers. To increase participation, the unawareness of its existence and usefulness needs to be addressed by informing the physicians of the possible benefits and subsequently encourage them to discuss the Web portal with their patients.

## Abbreviations

BDKT: Brief Diabetes Knowledge Test
DMSES: Diabetes Management Self-Efficacy Scale
DTSQ: Diabetes Treatment Satisfaction Questionnaire
PAID: Problem Areas in Diabetes
